# Supplementary material for: A Likelihood Approach for Real-Time Calibration of Stochastic Compartmental Epidemic Models
Source: PLoS Comput Biol. 2017 Jan 17;13(1):e1005257. doi: 10.1371/journal.pcbi.1005257 (PMC5240920; doi:10.1371/journal.pcbi.1005257)
Supplement: S1 File — (TAR.GZ) [file pcbi.1005257.s014.tar.gz › HSPH_Online-SI-Revision/PF08/n100-mild/n100-mild_PF2_table4.pdf]

1	newly 3 weeks spec	734.	5385.41	{5273., 5520.}	633.707	0.865459	newly 3 weeks cumul	3465.	17568.6	{17386., 17738.}	407.03	0.705017	newly 5 weeks spec	287.	4141.27	{4057., 4223.}	1342.95	1.15919	newly 5 weeks cumul	4194.	26485.	{26177., 26773.}	531.498	0.800356	newly 48 weeks cumul	4721.	45990.6	{44830., 46618.}	874.171	0.988597
2	newly 3 weeks spec	1022.	4383.52	{4264., 4526.}	328.916	0.632252	newly 3 weeks cumul	4122.	14691.3	{14411., 15040.}	256.411	0.551883	newly 5 weeks spec	367.	3239.01	{3136., 3355.}	782.564	0.945574	newly 5 weeks cumul	5105.	21728.4	{21313., 22275.}	325.63	0.628954	newly 47 weeks cumul	5925.	36231.7	{35553., 36904.}	511.505	0.786308
3	newly 3 weeks spec	703.	5190.46	{5071., 5286.}	638.33	0.868181	newly 3 weeks cumul	2922.	16963.4	{16666., 17199.}	480.541	0.7638	newly 5 weeks spec	387.	3993.38	{3834., 4111.}	931.881	1.01345	newly 5 weeks cumul	3733.	25559.9	{25171., 25932.}	584.701	0.835453	newly 50 weeks cumul	4191.	44412.7	{41824., 45220.}	959.715	1.02501
4	newly 3 weeks spec	301.	3390.74	{3292., 3487.}	1026.49	1.05162	newly 3 weeks cumul	2102.	13181.1	{12955., 13472.}	527.076	0.797273	newly 5 weeks spec	86.	2020.9	{1956., 2076.}	2249.88	1.37092	newly 5 weeks cumul	2358.	17830.3	{17540., 18173.}	656.161	0.878571	newly 54 weeks cumul	2427.	24257.8	{23890., 24735.}	899.496	0.999739
5	newly 3 weeks spec	828.	4413.9	{4297., 4537.}	433.08	0.726689	newly 3 weeks cumul	3560.	15132.2	{14924., 15386.}	325.061	0.628417	newly 5 weeks spec	386.	3103.73	{2998., 3198.}	704.075	0.905164	newly 5 weeks cumul	4530.	21971.	{21651., 22379.}	385.012	0.685718	newly 50 weeks cumul	5288.	34315.7	{33809., 34845.}	548.935	0.812167
6	newly 3 weeks spec	764.	4201.21	{4034., 4352.}	449.897	0.740103	newly 3 weeks cumul	3259.	14303.	{13939., 14623.}	338.878	0.642271	newly 5 weeks spec	315.	3063.54	{2916., 3181.}	872.552	0.987654	newly 5 weeks cumul	4097.	20981.	{20336., 21457.}	412.105	0.709263	newly 48 weeks cumul	4608.	34497.	{33123., 35820.}	648.633	0.874051
7	newly 3 weeks spec	276.	5159.32	{4983., 5302.}	1769.32	1.27156	newly 3 weeks cumul	1666.	19225.9	{18666., 19526.}	1054.02	1.06216	newly 5 weeks spec	53.	3221.11	{3060., 3328.}	5977.57	1.78352	newly 5 weeks cumul	1818.	26542.5	{25586., 27016.}	1359.98	1.16428	newly 53 weeks cumul	1893.	37207.8	{35803., 38023.}	1865.54	1.29337
8	newly 3 weeks spec	397.	4689.75	{4577., 4799.}	1081.3	1.07227	newly 3 weeks cumul	2114.	15906.5	{15717., 16169.}	652.434	0.876434	newly 5 weeks spec	163.	3416.04	{3323., 3512.}	1995.73	1.32112	newly 5 weeks cumul	2526.	23363.9	{23078., 23646.}	824.936	0.966062	newly 47 weeks cumul	2724.	38287.5	{37544., 38691.}	1305.56	1.14768
9	newly 3 weeks spec	565.	4030.8	{3948., 4106.}	613.416	0.853288	newly 3 weeks cumul	2870.	15320.5	{15165., 15460.}	433.814	0.727374	newly 5 weeks spec	200.	2448.26	{2374., 2512.}	1124.13	1.08773	newly 5 weeks cumul	3444.	20918.5	{20732., 21108.}	507.391	0.783455	newly 52 weeks cumul	3765.	28746.2	{28533., 29013.}	663.511	0.882805
10	newly 3 weeks spec	792.	3523.38	{3424., 3602.}	344.871	0.648123	newly 3 weeks cumul	3481.	11694.3	{11528., 11840.}	235.945	0.526224	newly 5 weeks spec	358.	2706.52	{2623., 2779.}	656.011	0.878371	newly 5 weeks cumul	4318.	17520.1	{17310., 17701.}	305.745	0.608204	newly 46 weeks cumul	4964.	31628.3	{31183., 31939.}	537.153	0.804172
11	newly 3 weeks spec	702.	4767.8	{4673., 4863.}	579.174	0.831928	newly 3 weeks cumul	3890.	16783.9	{16671., 16903.}	331.462	0.634936	newly 5 weeks spec	225.	3222.9	{3137., 3305.}	1332.4	1.15597	newly 5 weeks cumul	4534.	23966.7	{23791., 24118.}	428.599	0.72312	newly 50 weeks cumul	4756.	36009.1	{35675., 36290.}	657.13	0.879161
12	newly 3 weeks spec	1213.	3890.53	{3809., 4000.}	220.736	0.506032	newly 3 weeks cumul	4897.	13344.9	{13202., 13467.}	172.513	0.435357	newly 5 weeks spec	613.	2788.06	{2702., 2864.}	354.822	0.657699	newly 5 weeks cumul	6341.	19456.5	{19204., 19692.}	206.836	0.486868	newly 46 weeks cumul	8505.	31458.6	{31076., 31744.}	269.883	0.567993
13	newly 3 weeks spec	527.	4194.78	{4063., 4314.}	695.973	0.900774	newly 3 weeks cumul	2892.	15555.4	{15280., 15843.}	437.878	0.730626	newly 5 weeks spec	244.	2578.27	{2462., 2690.}	956.668	1.0237	newly 5 weeks cumul	3457.	21447.	{20964., 21939.}	520.392	0.792591	newly 51 weeks cumul	3655.	29394.2	{28541., 30490.}	704.22	0.905242
14	newly 3 weeks spec	361.	3508.3	{3404., 3620.}	871.828	0.987461	newly 3 weeks cumul	1836.	13377.1	{13145., 13623.}	628.601	0.862442	newly 5 weeks spec	115.	2138.96	{2067., 2224.}	1759.97	1.26932	newly 5 weeks cumul	2169.	18272.	{17952., 18601.}	742.416	0.925476	newly 53 weeks cumul	2408.	25341.3	{24763., 25861.}	952.38	1.0221
15	newly 3 weeks spec	612.	4565.69	{4482., 4660.}	646.028	0.872705	newly 3 weeks cumul	3189.	16556.2	{16437., 16698.}	419.167	0.715299	newly 5 weeks spec	228.	2955.28	{2867., 3031.}	1196.18	1.11255	newly 5 weeks cumul	3718.	23223.1	{23038., 23419.}	524.612	0.795599	newly 50 weeks cumul	4085.	33550.8	{33096., 34040.}	721.318	0.914481
16	newly 3 weeks spec	1144.	4222.35	{4107., 4336.}	269.087	0.567032	newly 3 weeks cumul	4245.	13301.5	{13057., 13538.}	213.344	0.495981	newly 5 weeks spec	573.	3521.67	{3411., 3631.}	514.602	0.788455	newly 5 weeks cumul	5611.	20715.4	{20266., 21080.}	269.192	0.567207	newly 46 weeks cumul	6762.	41080.9	{39590., 42013.}	507.526	0.783458
17	newly 3 weeks spec	602.	4617.46	{4461., 4790.}	667.02	0.884655	newly 3 weeks cumul	3587.	17377.3	{16942., 17857.}	384.451	0.685175	newly 5 weeks spec	237.	2767.82	{2667., 2875.}	1067.86	1.06721	newly 5 weeks cumul	4153.	23755.5	{23181., 24510.}	472.007	0.757324	newly 52 weeks cumul	4431.	31827.1	{31079., 32846.}	618.283	0.856218
18	newly 3 weeks spec	80.	4831.51	{4756., 4902.}	5939.39	1.78096	newly 3 weeks cumul	978.	18447.9	{18325., 18567.}	1786.29	1.2756	newly 5 weeks spec	21.	2905.98	{2838., 2987.}	13738.	2.141	newly 5 weeks cumul	1011.	25126.3	{24999., 25259.}	2385.29	1.39537	newly 56 weeks cumul	1018.	34279.3	{34135., 34423.}	3267.31	1.52728
19	newly 3 weeks spec	761.	4830.56	{4705., 4939.}	534.765	0.802536	newly 3 weeks cumul	3302.	15753.2	{15517., 15984.}	377.081	0.678559	newly 5 weeks spec	360.	3750.33	{3615., 3866.}	941.758	1.01762	newly 5 weeks cumul	4108.	23803.9	{23408., 24181.}	479.453	0.762984	newly 47 weeks cumul	4733.	42004.2	{41061., 42687.}	787.475	0.948111
20	newly 3 weeks spec	252.	5384.82	{5270., 5479.}	2036.83	1.32973	newly 3 weeks cumul	2135.	20767.6	{20518., 20997.}	872.719	0.987972	newly 5 weeks spec	37.	3188.85	{3130., 3256.}	8518.51	1.93536	newly 5 weeks cumul	2324.	28124.6	{27850., 28395.}	1110.18	1.08284	newly 56 weeks cumul	2336.	37815.8	{37360., 38189.}	1518.83	1.20918
21	newly 3 weeks spec	232.	4912.36	{4724., 5033.}	2017.4	1.32569	newly 3 weeks cumul	1801.	18849.7	{18420., 19287.}	946.625	1.01973	newly 5 weeks spec	64.	2770.93	{2665., 2862.}	4229.58	1.6363	newly 5 weeks cumul	1977.	25341.5	{24738., 25934.}	1181.82	1.10777	newly 56 weeks cumul	2067.	32526.7	{31729., 33310.}	1473.62	1.19683
22	newly 3 weeks spec	1379.	3856.89	{3584., 4067.}	179.687	0.446186	newly 3 weeks cumul	5215.	12781.7	{12125., 13293.}	145.095	0.38905	newly 5 weeks spec	709.	2948.94	{2661., 3171.}	315.929	0.618141	newly 5 weeks cumul	6926.	19134.2	{17988., 20035.}	176.267	0.440933	newly 46 weeks cumul	9186.	33884.6	{31114., 36131.}	268.872	0.566097
23	newly 3 weeks spec	467.	5217.91	{5063., 5333.}	1017.33	1.04808	newly 3 weeks cumul	3051.	18766.2	{18392., 19137.}	515.085	0.788877	newly 5 weeks spec	143.	3357.55	{3236., 3484.}	2247.94	1.37051	newly 5 weeks cumul	3445.	26354.6	{25775., 26880.}	665.009	0.88361	newly 51 weeks cumul	3590.	37441.8	{36487., 38222.}	942.947	1.01819
24	newly 3 weeks spec	99.	4078.9	{3965., 4173.}	4020.1	1.6148	newly 3 weeks cumul	1045.	14025.6	{13842., 14185.}	1242.16	1.12774	newly 5 weeks spec	29.	2921.49	{2836., 2997.}	9974.1	2.00307	newly 5 weeks cumul	1106.	20412.	{20149., 20634.}	1745.57	1.26607	newly 55 weeks cumul	1133.	33742.5	{33266., 34120.}	2878.15	1.47388
25	newly 3 weeks spec	1111.	4437.48	{4321., 4546.}	299.413	0.601338	newly 3 weeks cumul	4640.	14379.3	{14106., 14615.}	209.9	0.491179	newly 5 weeks spec	633.	3507.48	{3384., 3621.}	454.104	0.743452	newly 5 weeks cumul	6102.	21859.5	{21425., 22223.}	258.235	0.554126	newly 46 weeks cumul	7554.	40565.1	{39774., 41484.}	437.002	0.729917
26	newly 3 weeks spec	720.	4480.88	{4370., 4602.}	522.344	0.793948	newly 3 weeks cumul	3694.	15422.5	{15209., 15675.}	317.501	0.620631	newly 5 weeks spec	332.	3188.04	{3101., 3292.}	860.253	0.982256	newly 5 weeks cumul	4491.	22420.7	{22143., 22770.}	399.237	0.698279	newly 48 weeks cumul	5251.	36098.9	{35488., 36726.}	587.467	0.837194
27	newly 3 weeks spec	1036.	4412.75	{4299., 4519.}	325.941	0.629264	newly 3 weeks cumul	4214.	14422.8	{14223., 14645.}	242.258	0.534327	newly 5 weeks spec	467.	3448.65	{3350., 3537.}	638.469	0.868234	newly 5 weeks cumul	5302.	21795.1	{21518., 22170.}	311.073	0.613895	newly 46 weeks cumul	6355.	39800.6	{39052., 40388.}	526.288	0.796728
28	newly 3 weeks spec	1038.	4323.99	{4165., 4610.}	316.569	0.619408	newly 3 weeks cumul	4164.	13969.7	{13637., 14710.}	235.488	0.525516	newly 5 weeks spec	683.	3436.2	{3284., 3784.}	403.104	0.701112	newly 5 weeks cumul	5692.	21302.9	{20741., 22679.}	274.261	0.572943	newly 47 weeks cumul	8123.	39483.5	{37788., 43868.}	386.07	0.686049
29	newly 3 weeks spec	584.	5037.44	{4931., 5120.}	762.575	0.935748	newly 3 weeks cumul	2514.	16252.6	{16091., 16399.}	546.483	0.810545	newly 5 weeks spec	328.	3916.91	{3824., 4007.}	1094.18	1.077	newly 5 weeks cumul	3268.	24661.4	{24418., 24885.}	654.632	0.877723	newly 49 weeks cumul	4524.	43224.2	{42810., 43574.}	855.442	0.980195
30	newly 3 weeks spec	466.	3949.38	{3865., 4034.}	747.506	0.92808	newly 3 weeks cumul	2784.	15543.3	{15415., 15665.}	458.307	0.746861	newly 5 weeks spec	120.	2234.9	{2145., 2310.}	1762.42	1.26992	newly 5 weeks cumul	3116.	20757.3	{20573., 20922.}	566.151	0.82356	newly 51 weeks cumul	3221.	26843.3	{26542., 27120.}	733.383	0.920821
31	newly 3 weeks spec	874.	5145.76	{5025., 5285.}	488.76	0.769852	newly 3 weeks cumul	3903.	16867.4	{16633., 17133.}	332.166	0.635617	newly 5 weeks spec	482.	3874.85	{3763., 3983.}	703.911	0.905107	newly 5 weeks cumul	4981.	25270.9	{24946., 25674.}	407.345	0.70527	newly 48 weeks cumul	5893.	42164.9	{41419., 42771.}	615.508	0.85457
32	newly 3 weeks spec	1191.	3835.64	{3719., 3932.}	222.052	0.507829	newly 3 weeks cumul	4640.	12801.2	{12656., 12981.}	175.888	0.440711	newly 5 weeks spec	844.	2888.29	{2782., 2986.}	242.214	0.534122	newly 5 weeks cumul	6529.	19038.2	{18770., 19280.}	191.594	0.464749	newly 46 weeks cumul	9247.	33055.8	{32338., 33699.}	257.476	0.553125
33	newly 3 weeks spec	62.	5680.52	{5584., 5772.}	9062.13	1.96195	newly 3 weeks cumul	424.	21664.5	{21517., 21813.}	5009.55	1.70838	newly 5 weeks spec	6.	3406.72	{3325., 3492.}	56678.7	2.75412	newly 5 weeks cumul	438.	29507.4	{29325., 29679.}	6636.86	1.82845	newly 56 weeks cumul	438.	39988.	{39799., 40190.}	9029.69	1.96045
34	newly 3 weeks spec	175.	3814.89	{3713., 3902.}	2079.94	1.33834	newly 3 weeks cumul	1326.	14647.7	{14465., 14845.}	1004.65	1.04318	newly 5 weeks spec	25.	2312.61	{2230., 2389.}	9150.44	1.96596	newly 5 weeks cumul	1434.	19938.1	{19654., 20283.}	1290.39	1.14308	newly 52 weeks cumul	1519.	27433.	{26994., 28089.}	1705.99	1.25663
35	newly 3 weeks spec	0.1	6349.03	{6243., 6456.}	6.34893×10^{6}	4.80267	newly 3 weeks cumul	47.	23660.9	{23491., 23790.}	50242.2	2.70193	newly 5 weeks spec	0.1	3876.26	{3787., 3953.}	3.87616×10^{6}	4.58834	newly 5 weeks cumul	47.	32534.1	{32396., 32679.}	69121.4	2.84024	newly 56 weeks cumul	47.	44654.1	{44483., 44815.}	94908.6	2.97776
36	newly 3 weeks spec	307.	6147.54	{6040., 6290.}	1902.46	1.30152	newly 3 weeks cumul	1782.	22275.8	{22098., 22463.}	1150.04	1.09692	newly 5 weeks spec	117.	3925.15	{3824., 4014.}	3254.83	1.5256	newly 5 weeks cumul	2037.	31153.3	{30881., 31392.}	1429.37	1.1845	newly 56 weeks cumul	2462.	44340.6	{43879., 44664.}	1701.	1.2555
37	newly 3 weeks spec	611.	4403.64	{4273., 4488.}	620.727	0.857699	newly 3 weeks cumul	3063.	15432.4	{15290., 15587.}	403.831	0.702268	newly 5 weeks spec	219.	3056.95	{2934., 3149.}	1295.87	1.1447	newly 5 weeks cumul	3644.	22187.5	{21847., 22424.}	508.877	0.784506	newly 49 weeks cumul	4006.	34255.5	{33547., 34904.}	755.104	0.931935
38	newly 3 weeks spec	40.	5183.25	{5046., 5316.}	12858.1	2.11246	newly 3 weeks cumul	901.	19908.1	{19610., 20205.}	2109.56	1.34427	newly 5 weeks spec	6.	3084.46	{3005., 3166.}	51307.7	2.71093	newly 5 weeks cumul	919.	27016.5	{26621., 27446.}	2839.77	1.46828	newly 56 weeks cumul	919.	36523.6	{35836., 37102.}	3874.27	1.59922
39	newly 3 weeks spec	244.	4169.42	{4087., 4253.}	1608.78	1.23264	newly 3 weeks cumul	1717.	15859.6	{15706., 16020.}	823.679	0.965509	newly 5 weeks spec	134.	2511.51	{2436., 2580.}	1774.26	1.2727	newly 5 weeks cumul	1991.	21626.3	{21431., 21855.}	986.201	1.0359	newly 53 weeks cumul	2147.	29432.	{29141., 29685.}	1270.84	1.13697
40	newly 3 weeks spec	154.	4070.57	{3984., 4148.}	2543.23	1.42208	newly 3 weeks cumul	1047.	16010.	{15893., 16136.}	1429.13	1.18444	newly 5 weeks spec	17.	2365.93	{2302., 2426.}	13817.2	2.14346	newly 5 weeks cumul	1131.	21475.4	{21351., 21590.}	1798.8	1.27847	newly 55 weeks cumul	1136.	28596.6	{28431., 28735.}	2417.3	1.40093
41	newly 3 weeks spec	226.	4669.81	{4588., 4758.}	1966.29	1.31514	newly 3 weeks cumul	1612.	18137.2	{18011., 18274.}	1025.13	1.05119	newly 5 weeks spec	99.	2760.51	{2702., 2806.}	2688.39	1.44529	newly 5 weeks cumul	1842.	24505.7	{24348., 24636.}	1230.39	1.12397	newly 56 weeks cumul	1901.	32914.4	{32755., 33078.}	1631.42	1.2384
42	newly 3 weeks spec	285.	4572.95	{4476., 4670.}	1504.54	1.2053	newly 3 weeks cumul	1860.	17373.6	{17207., 17549.}	834.066	0.970363	newly 5 weeks spec	90.	2759.88	{2705., 2810.}	2966.53	1.48659	newly 5 weeks cumul	2101.	23720.4	{23547., 23919.}	1029.	1.05269	newly 53 weeks cumul	2198.	32413.	{32186., 32652.}	1374.66	1.16868
43	newly 3 weeks spec	344.	4676.5	{4595., 4769.}	1259.45	1.13332	newly 3 weeks cumul	2328.	17880.8	{17765., 18042.}	668.076	0.885396	newly 5 weeks spec	97.	2802.95	{2745., 2858.}	2789.64	1.46077	newly 5 weeks cumul	2595.	24329.	{24208., 24465.}	837.534	0.971983	newly 56 weeks cumul	2707.	33148.2	{32991., 33296.}	1124.53	1.08797
44	newly 3 weeks spec	177.	4424.6	{4342., 4512.}	2399.77	1.39786	newly 3 weeks cumul	1333.	17350.7	{17217., 17496.}	1201.63	1.11448	newly 5 weeks spec	67.	2582.22	{2534., 2640.}	3754.06	1.58584	newly 5 weeks cumul	1521.	23333.3	{23171., 23495.}	1434.07	1.18584	newly 55 weeks cumul	1548.	31133.5	{30952., 31358.}	1911.21	1.30345
45	newly 3 weeks spec	708.	4557.94	{4437., 4683.}	543.777	0.80863	newly 3 weeks cumul	3626.	15739.9	{15499., 15949.}	334.085	0.637539	newly 5 weeks spec	308.	3171.38	{3080., 3275.}	929.669	1.01246	newly 5 weeks cumul	4438.	22746.6	{22464., 23066.}	412.541	0.709679	newly 51 weeks cumul	4856.	34822.3	{34175., 35399.}	617.098	0.85547
46	newly 3 weeks spec	683.	4895.51	{4722., 5060.}	616.766	0.855216	newly 3 weeks cumul	3807.	18434.7	{17872., 18894.}	384.231	0.684951	newly 5 weeks spec	255.	2915.58	{2813., 3009.}	1043.36	1.05802	newly 5 weeks cumul	4509.	25166.8	{24445., 25738.}	458.146	0.746659	newly 52 weeks cumul	4663.	33481.5	{32640., 34195.}	618.025	0.856079
47	newly 3 weeks spec	364.	4279.09	{4159., 4396.}	1075.57	1.07011	newly 3 weeks cumul	2257.	16250.8	{15994., 16680.}	620.016	0.857267	newly 5 weeks spec	75.	2607.12	{2511., 2742.}	3376.16	1.5408	newly 5 weeks cumul	2514.	22204.5	{21796., 22951.}	783.234	0.945973	newly 52 weeks cumul	2640.	30696.3	{30085., 31881.}	1062.74	1.06532
48	newly 3 weeks spec	674.	4778.45	{4675., 4880.}	608.969	0.850576	newly 3 weeks cumul	3437.	15491.9	{15348., 15609.}	350.738	0.653915	newly 5 weeks spec	214.	3757.28	{3666., 3845.}	1655.74	1.24439	newly 5 weeks cumul	4062.	23526.7	{23358., 23670.}	479.189	0.762814	newly 47 weeks cumul	4384.	42843.9	{42531., 43211.}	877.279	0.990011
49	newly 3 weeks spec	230.	4556.1	{4436., 4661.}	1880.91	1.29678	newly 3 weeks cumul	1489.	16825.	{16557., 17026.}	1029.95	1.05304	newly 5 weeks spec	69.	2792.4	{2700., 2873.}	3946.96	1.607	newly 5 weeks cumul	1667.	23217.3	{22809., 23521.}	1292.76	1.14385	newly 53 weeks cumul	1729.	31654.7	{31001., 32112.}	1730.81	1.26261
50	newly 3 weeks spec	221.	3640.47	{3553., 3719.}	1547.27	1.21669	newly 3 weeks cumul	1420.	14274.5	{14108., 14460.}	905.243	1.00225	newly 5 weeks spec	59.	2112.66	{2046., 2180.}	3480.78	1.55382	newly 5 weeks cumul	1550.	19166.6	{18985., 19407.}	1136.55	1.09219	newly 54 weeks cumul	1593.	25477.4	{25188., 25830.}	1499.34	1.20391
